# Lycorine inhibits glioblastoma multiforme growth through EGFR suppression

**DOI:** 10.1186/s13046-018-0785-4

**Published:** 2018-07-17

**Authors:** Jia Shen, Tao Zhang, Zheng Cheng, Ni Zhu, Hua Wang, Li Lin, Zexia Wang, Haotian Yi, Meichun Hu

**Affiliations:** 1 Research Center of Basic Medical Sciences, School of Basic Medical Sciences, Hubei University of Science and Technology, 88 Xianning Avenue, Xianning, 437000 China; 2 Institute of Biochemistry and Cell Biology, Shanghai Institutes for Biological Sciences, Chinese Academy of Sciences, Shanghai, 200031 China; 3 Cancer Center, Sanford Burnham Prebys Medical Discovery Institute, La Jolla, California, 92037 USA

**Keywords:** Lycorine, Glioblastoma multiforme, EGFR signaling pathway, Wild type EGFR, EGFRvIII, Tumor growth

## Abstract

**Background:**

Lycorine has been revealed to inhibit the development of many kinds of malignant tumors, including glioblastoma multiforme (GBM). Although compelling evidences demonstrated Lycorine’s inhibition on cancers through some peripheral mechanism, in-depth mechanism studies of Lycotine’s anti-GBM effects still call for further exploration. Epidermal Growth Factor Receptor (EGFR) gene amplification and mutations are the most common oncogenic events in GBM. Targeting EGFR by small molecular inhibitors is a rational strategy for GBM treatment.

**Methods:**

The molecular docking modeling and in vitro EGFR kinase activity system were employed to identify the potential inhibitory effects of Lycorine on EGFR. And the Biacore assay was used to confirm the direct binding status between Lycorine and the intracellular EGFR (696–1022) domain. In vitro assays were conducted to test the suppression of Lycorine on the biological behavior of GBM cells. By RNA interference, EGFR expression was reduced then cells underwent proliferation assay to investigate whether Lycorine’s inhibition on GBM cells was EGFR-dependent or not. RT-PCR and western blotting analysis were carried out to investigate the underlined molecular mechanism that Lycorine exerted on EGFR itself and EGFR signaling pathway. Three different xenograft models (an U251-luc intracranially orthotopic transplantation model, an EGFR stably knockdown U251 subcutaneous xenograft model and a patient-derived xenograft model) were performed to verify Lycorine’s therapeutic potential on GBM in vivo.

**Results:**

We identified a novel small natural molecule Lycorine binding to the intracellular EGFR (696–1022) domain as an inhibitor of EGFR. Lycorine decreased GBM cell proliferation, migration and colony formation by inducing cell apoptosis in an EGFR-mediated manner. Furthermore, Lycorine inhibited the xenograft tumor growths in three animal models in vivo. Besides, Lycorine impaired the phosphorylation of EGFR, AKT, which were mechanistically associated with expression alteration of a series of cell survival and death regulators and metastasis-related MMP9 protein.

**Conclusions:**

Our findings identify Lycorine directly interacts with EGFR and inhibits EGFR activation. The most significant result is that Lycorine displays satisfactory therapeutic effect in our patient-derived GBM tumor xenograft, thus supporting the conclusion that Lycorine may be considered as a promising candidate in clinical therapy for GBM.

## Background

Gliomas are the most common brain tumor in adults, accounting for about 70% of primary neoplasms of the central nervous system (CNS). High-grade gliomas, especially the glioblastoma multiforme (GBM), is the most common and progressive type during all intracranial cancers [1]. About 90% of GBMs are classified as primary and associated with dismal prognosis that typically appears suddenly in patients. On one hand, such lesions affect mainly the elderly (mean age 62 years), have rapid evolution (less than 3 months) and no clinical or histopathological evidence of precursor lesions [2]. On the other hand, secondary GBMs affect younger individuals (average age 45 years) and progress slowly from a lower degree of diffuse astrocytoma. Current therapeutic strategies for GBM include surgical resection, followed by radiotherapy and chemotherapy [3–5]. Despite such aggressive multimodal therapy, the median survival of GBM is still poor [6]. The high mortality rate results from the universal resurgence of tumors post-treatment, which occurs due to infiltrating tumor cells that escape initial surgery and exhibit profound resistance to irradiation and current chemotherapy treatments [7]. With the increasing number of cancer-related mortality, identification of novel tractable targets for improved therapeutics and development for novel drugs that can radically cure GBM are desperately needed.

Genomic and proteomic analyses have identified a number of key oncogenic drivers of GBM tumorigenesis and therapeutic resistance, including receptor tyrosine kinases (RTKs) [8]. In particular, genomic alteration of the epidermal growth factor receptor (EGFR) is present in approximately half of all GBMs [9, 10]. EGFR plays an important role in various tumors including GBM. It is the most frequently amplified gene in GBM, while its expression in normal brain tissue is either undetectable or extremely low [11]. The most common genetic aberration associated with malignant glioma is amplification of EGFR, with a frequency of about 50%. Amplifications and rearrangements of EGFR are highly indicative of high-grade gliomas, with a worse prognosis than estimated from just histopathologic grading [12]. EGFR activation leads to autophosphorylation of several key tyrosine residues triggering several intracellular downstream signaling pathways including the Ras/Raf/MEK/ERK pathway, the PLCγ -PKC pathway and the PI3K/AKT pathway, resulting in cell proliferation, motility and survival [13]. Within such a large proportion of EGFR genomic alterations, approximately 20–40% of them harbor EGFR variant III (EGFRvIII) mutant, which contains a deletion of exons 2–7 in the extracellular ligand-binding domain [14, 15]. EGFRvIII induces the receptor tyrosine kinase activation in both a cell autonomous and nonautonomous manner, thus results in a ligand-independent mutant and shows constitutive activation in the absence of ligand to activate the tumor-promoting signaling pathways [16].

The fact that EGFR functions one of the most vital factors to promote gliomas has attracted many investigations of EGFR inhibitors, aiming to promote apoptosis of cancer cells, or to increase tumor sensitivity to possible adjuvant therapies. However, the successful application of EGFR-targeted therapy for the treatment of GBM has proven to be very challenging. Many GBM patients do not respond to these therapies and eventually show drug resistance and disease progression [16]. To screen and develop novel inhibitors that target both wild type EGFR and EGFRvIII to impair GBM malignant tumor cell biology could be therapeutically beneficial either as single agents or in combination with other chemotherapy agents in gliomas therapy.

Lycorine is a pyrrolo[de]phenanthridine ring-type alkaloid extracted from Amaryllidaceae genera and possesses various biological effects including anti-tumor [17], antiviral [18], antimalarial [19], and antiinflammation [20]. Several studies have shown that Lycorine exhibits selective cytotoxicity effects on leukemia, cervical cancer, multiple myeloma, prostate cancer, hepatocellular carcinoma, bladder cancer and breast cancer [21–24]. And the anti-cancer effect of Lycorine on glioblastoma, even in drug-resistant glioblastoma, were also reported in some studies [22, 25–28] . Typically, Lycorine triggered multiple myeloma KM3 cells’ apoptosis through the activated molecular signaling in mitochondrial and death receptor-mediated apoptotic pathways [29]. Lycorine also induced cell-cycle arrest in myelogenous leukemia K562 cells via HDAC inhibition [30]. Additionally, Lycorine has been reported to exhibit anti-proliferative, apoptosis-inducing, and anti-invasive properties in prostate cancer associated with the JAK-STAT signaling pathway. Lycorine promoted autophagy and apoptosis via TCRP1/Akt/mTOR axis inactivation in human hepatocellular carcinoma [31]. Lycorine induced apoptosis of bladder cancer T24 cells by inhibiting phospho-Akt and activating the intrinsic apoptotic cascade [32]. Lycorine could impair human glioblastoma U373 cell migration by increasing cellular actin cytoskeleton rigidity possibly through modulating the Rho/Rho kinase/LIM kinase/cofilin signaling pathway [22]. Furthermore, Lycorine possessed favorable in vitro anti-proliferative activity through cytostatic rather than cytotoxic effects towards apoptosis-resistant U373 cells because of its structural features of a C-ring and C/D-ring junction, which was essential for its biological activities [28]. Besides, a structure-activity relationship (SAR) analysis of Lycorine with its intracellular targets revealed Lycorine’s both anti-proliferative and apoptosis-inducing activities in human glioblastoma apoptosis-resistant T98G cells and in human glioblastoma apoptosis-sensitive HS683 cells. Lycorine’s C1, C2-hydroxyls provided a superior binding pose with the pocket a, the guanosine triphosphate (GTP) binding site, of its target protein eEF1A elucidated by the molecular docking results [26].

Although accumulating evidences demonstrated Lycorine’s inhibition effects on cancers including glioblastoma, through some peripheral mechanism such as the currently most acceptable mode of Lycorine’s action of its inhibition on DNA and protein biosynthesis in cancer cells, or through some other complex unrevealed way, in-depth mechanism studies of Lycotine’s anti-GBM effects still call for further exploration. Researches to determine Lycorine’s underlying mechanisms besides abovementioned in cancer cells are warranted. A wealthy X-ray structural information of Lycorine in complex with eukaryotic ribosome had also been found associated with the inhibition of the elongation cycle during the protein translation process to alter cell proliferation and protein synthesis. Lycorine adopted a special conformation within the pocket region in the A-site of the peptidyl transferase center of ribosomes, which suggested that the dioxol-pyrroline group of Lycorine might be a recognition motif for the binding with its target complex proteins. Lycorine’s X-ray structure-based drug design may highlight general principles for its targeting and facilitate its new therapeutics design, thus serving as a tool to guide Lycorine’s future drug research and development [33]. Those abovementioned signals, such as JAK, STAT, AKT and mTOR, involved in Lycorine’s inhibition on many kinds of cancer types, were all downstream pathway signals relative to tyrosine kinase. This prompt us to form the hypothesis that the underling in-depth mechanism of Lycorine’s inhibition on GBM cancer may fundamentally correlate with some classical tyrosine kinase pathway, for example, the EGFR signaling pathway.

In accordance with existing researches and the X-ray structure of Lycorine, we identify Lycorine as a novel inhibitor directly targeting EGFR through molecular docking assay and Biacore assay, and our findings propose a fundamental in-depth mechanism of Lycorine’s suppression on GBM growth. To our knowledge, investigations of Lycorine’s interaction with EGFR have not been described in previous literature. We present in this current study that Lycorine inhibits proliferation and migration of various GBM cell lines,including cells holding wild type EGFR amplification and EGFRvIII, and induces cell apoptosis and cell death. In vivo experiments show that intraperitoneal administration of Lycorine reduces tumor growth in U251-luc intracranially orthotopic transplantation model, EGFR stable knockdown abates Lycorine’s treatment effect in mice subcutenous xenografts, and in patient-derived xenograft model Lycorine exhibits impressive efficacy with no obvious toxicity. Lycorine inhibits the activation of EGFR signaling and multiple EGFR downstream targets, such as AKT, ERK, mTOR, cyclin D1, Bcl-2, Bcl-xL, and matrix metalloproteinase 9 (MMP9). In conclusion, our findings suggest that Lycorine is a new small molecule targeting EGFR thus may be a potential therapeutic in combating GBM.

## Methods

### Cell culture, animals and reagents

Human GBM cells including U87 (wild type EGFR), LN229 (wild type EGFR amplification), U251 (wild type EGFR amplification), A172 (EGFRvIII mutant), Gli36vIII (EGFRvIII mutant), GBM6 (wild type EGFR and EGFRvIII co-existing), healthy normal human IMA2.1 astrocytes and human embryonic kidney cell line 293 T were all purchased from American Type Culture Collection. The patient-derived cell line was primarily separated from an advanced GBM patient’s *in sute* tumor in Xianning central hospital, the first affiliated hospital of Hubei University of Science and Technology (Xianning China), with the patient’s informed consent. IMA2.1 astrocytes, U87 and U251 cells were cultured in Dulbecco’s Modified Eagle Medium (Gibco). LN229, A172, Gli36vIII and GBM6 cells were maintained in RPMI-1640 medium (Gibco). Both mediums were supplemented with 10% fetal bovine serum (Wisent). In addition, U251 cells were transfected with pGL4 vector (Promega) which stably expressed luciferase and selected in G418 to screen the stable U251-luc cell line. All cells were incubated at 37 °C of 5% humidified CO_2_. Nude mice BALB c/c were purchased from Beijing Vital River Laboratory Animal Technology Co., Ltd. All animal experimental protocols were approved by the Animal Investigation Committee of Hubei University of Science and Technology and Sanford/Burnham/Prebys Medical Discovery Institute. Lycorine (purity > 98%) was purchased from Shanghai Winherb Medical Science. Gefitinib was purchased from Shanghai Alis Chemicals Co. Ltd. Antibodies used to detect the protein expression levels of in vitro human GBM whole cell lysates for phospho-EGFR (Tyr1068) (#3777), EGFR (#4267), p-AKT (#4060), p-ERK (#9101), p-mTOR (#2971), p27 (#3688), p21 (#2946), Bcl-2 (#4223), Cyclin D1 (#2078), MMP9 (#13667) were all ordered from Cell Signaling Technology (Danvers, MA). Antibodies for human β-actin (#A5441) was from Sigma-Aldrich Co (St. Louis, MO). Ltd. Antibodies against PARP (sc-136,208), cleaved PARP (sc-56,196), Caspase 3 (sc-271,028) were all purchased from Santa Cruz. The anti-GST antibody was purchased from GE Healthcare (GE27–4577-01). Antibodies used to detect the protein expression levels of in vivo xenografts that dissected from tumor-bearing mice for phospho-EGFR (#4404) and EGFR (#4405) were purchased from Cell Signaling Technology (Danvers, MA). Antibodies for human β-actin (ab115777), GFAP (ab33922), Bcl-XL (ab15274), cleaved Caspase 3 (ab208003), Ki-67(ab92742) and PCNA (ab220208) were all from Abcam. All the antibodies used to detect in vivo proteins could specifically react to human proteins with nonspecific immunity reaction to mouse proteins.

### Molecular docking modeling assay

The X-ray crystal structure of EGFR was obtained from the Protein data bank ((PDB ID: 5FED, EGFR kinase domain in complex with a covalent aminobenzimidazole inhibitor) website (http://www.rcsb.org/). The structures of the ligands were built and energy minimized using the Chemoffice software package (Cambridge). We used AutoDock Toolkit developed by the Scripps Research Institute and Olson lab for free for docking experiments. All of the water molecules were removed before the experiments so that our experiments were performed under non-aqueous conditions. The primary ligand bound to the binding pocket was the chosen conformation for the active site. The picture was prepared using Pymol 1.2R2 version.

### In vitro EGFR kinase assay

The half maximal inhibitory concentration (IC_50_) values of Lycorine and positive control Gefitinib against EGFR kinase activity were carried out using the Promega Kinase-Glo kit (Promega, Mannheim, Germany) according to the manufacturer’s protocol in the presence of 600 nM ATP. Data were presented as means and 95% confidence intervals (CIs) from three independent experiments.

### Biacore assay for surface plasmon resonance (SPR) analysis

Firstly the human EGFR (696–1022) domain recombinant fusion protein was expressed in *Escherichia coli* (*E.coli*) BL21 (DE3). In detail, BL21 (DE3) was transformed with pGEX4T-1- EGFR (696–1022) plasmid which was constructed through molecular cloning methods in our laboratory. When the OD value reached about 0.6, the *E.coli* was transfected and induced to express recombinant fusion protein by adding 0.5 mM Isopropyl β-D-1-thiogalactopyranoside (IPTG). The soluble protein was obtained by sonication and centrifugation, then incubated with Glutathione-Sepharose beads (GE Healthcare), and eluted with glutathione. Fusion protein was further concentrated with ultrafiltration centrifuge tube and its concentration was determined. Then the SPR analysis was conducted with a Biacore T200 instrument (GE Healthcare) with CM5 sensor chip. In order to capture EGFR (696–1022) with GST tag, GST antibody was immobilized in parallel-flow channels of CM5 sensor chip. To test the interaction between Lycorine and EGFR, a series concentrations of Lycorine were injected into the flow system. Experiments was conducted with PBS buffer and the dissociation time was 60 s. Since Lycorine was dissolved in PBS with 5% DMSO, solvent correction assay was performed to adjust the results.

### Western blotting analysis

For detecting the effects of Lycorine’s long time treatment on the expression of EGFR as well as phosphorylation of EGFR and its downstream signaling pathways, U251 cells were pretreated with 100 ng/mL human recombinant protein EGF (Thermo Fisher scientific, PHG0311) for 6 h, then treated with Lycorine for indicated concentration for another 24 h; for detecting the effects of Lycorine’s short period treatment on the expression of EGFR and phosphorylation of EGFR, U251 cells were pretreated with 100 ng/mL human recombinant protein EGF for indicated time course (0, 15, 30, 45 and 60 min), then treated with 25 μM Lycorine for another 1 h; to prove that Lycorine inhibited the EGF-dependent activation of EGFR kinase phosphorylation, U251 cells were initially pretreated with or without 25 μM Lycorine for 1 h to allow Lycorine enter the cells, then followed by 100 ng/mL EGF treatment for 0, 15, 30, 45 and 60 min (Lycorine was maintained during the EGF-treated time course), and EGF-dependent EGFR phosphorylation was measured; for detecting the expression level of EGFR knockdown, the U251 parental, shControl and shEGFR cells were cultured in 6-well plates and whole cell lysate protein were extracted then subjected to the western blotting analysis; for detecting in vivo protein level in xenografts, the in vivo xenografe tissues were grinded in liquid nitrogen, then cells samples or tissue samples were lysed in RIPA buffer, respectively. Protein concentration was determined using a Bicinchoninic acid assay (Thermo Scientific). Protein samples were run on 8 to 12% SDS-PAGE gels and transferred to polyvinylidene difluoride membranes (Gibco) as detailed before [34, 35]. The membranes were incubated overnight using specific antibodies. The signals were visualized via the Odyssey Western blotting detection system.

### Semi-quantitative real-time polymerase chain reaction analysis

To detect human EGFR mRNA expression in glioma cells, RNA was extracted from the cell lines with RNeasy Mini Kit (Qiagen, Hilden, Germany) and quantified with NanoDrop (Thermo Fisher, Wilmington, DE). Reverse transcripts were produced using M-MLV reverse transcriptase (Invitrogen, Grand Island, NY), and PCR was conducted with GoTaq® Flexi DNA Polymerase (Promega, Madison, WI). The forward primer for EGFR is 5’-TGACTCCGTCCAGTATTGATCG-3′, and the reverse primer is 5’-GCCCTTCGCACTTCTTACACTT-3′. The forward primer for human EGF is 5’-ACCAACACGGAGGGAGGCTACAA-3′ and the reverse primer is 5’-GCGGTCCACGGATTCAACATACA-3′. Human GAPDH served as a loading control. Human GAPDH forward primer: 5’-GAAGGTGAAGGTCGGAGTCA-3′, reverse primer: 5’-TTGAGGTCAATGAAGGGGTC-3′ [36]. The PCR reaction parameters are 95 °C 5 min, 35 cycles at 95 °C 40 s, 55 °C 40 s, 72 °C 1 min, and final extension at 72 °C for 10 min. In vivo xenografe tissues were grinded in liquid nitrogen and the RNA was also isolated for RT-PCR analysis.

### SRB cell viability assay

SRB cell viability assays were performed by stained with Sulforhodamine B. Briefly, 5000 cells per well were seeded in 96-well plates as detailed before [37, 38]. After 24 h, cells were exposed to different concentrations of Lycorine for 48 h. Cells were fixed with 10% trichloroacetic acid for 1 h at 4 °C, washed five times with flowing water, and air-dried, then stained with 50 μL 0.4% (*w*/*v*) SRB for 20 min at room temperature, washed five times with 1% acetic acid, and air-dried. 100 μL 10 mM Tris was added per well, and absorbance was measured at 515 nm. For the detection of EGFR RNA-interference stable cells’ viability, the U251 parental, shControl and shEGFR cells were cultured in 96-well plates for indicated days (0, 1, 3, 5 and 7) with no Lycorine treatment, and their cell viabilities were analyzed, respectively.

### Migration assay

Cells were allowed to grow into Trans well /Boyden chambers (8 μm; BD Biosciences). Serum-starved U251 cells (5 × 10^4^ cells) in 100 μL medium with 0.5% FBS were pretreated with Lycorine (from 0 μM to 10 μM) for 30 min. Cells were then seeded on the upper chamber of Transwell and migrated to the lower chamber with 600 μL medium. After 5 to 7 h incubation, non-migrated cells were removed with cotton swabs, and migrated cells were fixed with cold 3.7% paraformaldehyde and stained with 0.1% crystal violet. Images were taken with an inverted microscope (Olympus; magnification, × 100), and migrated cells in 4 random fields were quantified by manual counting.

### Colony formation assay

Cells were trypsinized and seeded 2000 per well in 6-well plates and allowed to attach overnight, then exposed to different concentration of Lycorine for 7 days. After being fixed with 4% paraformaldehyde for 20 min, cells were stained with 0.2% crystal violet as detailed before [39]. The morphology of cell colonies was recorded with photo imaging and the number of cell colonies were calculated and analyzed as the ratio of the number and diameters of treated samples to untreated sample.

### Construction of stable EGFR knockdown cell line

A specific EGFR shRNA Lentiviral particle containing EGFR gene interfere sequence was purchased from santa cruz biotechnology (sc-108,050-SH). This interfere sequences were linked to pLL3.7 lentiviral expression vector and co-transfected into 293 T cells along with the packaging plasmids (pGag/Pol, pRev and pVSV-G) by Lipofectamine 2000 (Invitrogen). The titer and infection efficiency were determined by observing the expression of GFP under fluorescence microscopy. With appropriate multiplicity of infection and several days of screening with puromycin, U251 cells were infected by lentivirus and the stable knockdown cells were screened out, labeled as shEGFR. And the empty plasmid containing control shRNA was simultaneously constrcted and labelled as shControl. These two U251 stable cell lines were employed for further in vitro cell proliferation assay and in vivo subcutaneous xenograft assay.

### U251-luciferase cell orthotropic transplantation xenograft model

U251-luc intracranially orthotopic transplantation model were performed to verify Lycorine’s therapeutic potential on GBM in vivo. Nude BALB c/c mice were anesthetized and fixed in a stereotactic apparatus, a burr hole was drilled 2 mm lateral and 1 mm anterior to the bregma to a depth of 3.25 mm, and 5 × 10^5^ U251-luc cells in 10 μL PBS were implanted. 7 days later, based on photon flux indexes detected by Xenogen IVIS 2000 Luminal Imager(PerkinElmer, Waltham, MA) with living image software (PerkinElmer), all mice bearing tumor were divided into three groups (*n* = 10 per group) randomly and the luminal photos were taken and photon flux indexes which could represent the orthotopic tumor sizes were recorded every 10 days. Lycorine (10 mg/kg/day per mouse and 20 mg/kg/day per mouse) was injected intraperitoneally every day. The control group was treated with DMSO. 40 days later, mice were sacrificed, and tumors in brain substances were removed and bioluminescence imaging were recorded. The growth rate curve of the tumor xenograft was evaluated by determining the photon flux indexes. GBM tumor xenografts were fixed and prepared for immunohistochemistry.

### U251 shEGFR subcutaneous xenograft model

U251 shEGFR stable cell lines was successfully constructed as above mentioned. For testing the growth rate difference between U251 shControl and shEGFR in vivo without Lycorine treatment, 7 × 10^6^ cells per mouse were inoculated into nude BALB c/c mice on the right back sides for indicated time. The beginning day of cell inoculation was defined as day 0 and tumors were allowed to grow for 32 days. Phenotype of tumor-bearing nude mice and their xenografts were taken photos at an interval of 8 days, and the growth curve of U251 shControl and shEGFR after their inoculation from day 0 to day 32 were analyzed according to tumor volumes calculated every 4 days, respectively. To detect Lycorine’s in vivo effects on GBM growth was dependent or independent of EGFR expression, we used nude mice to conduct the same subcutaneous xenograft assay again with Lycorine administration. U251 shControl cells and U251 shEGFR cells (7 × 10^6^ cells per mouse) were separately inoculated subcutaneously on the right back sides of the mice in correspondent group. When the tumors reached about 100 mm^3^ after cell inoculation for 12 days, mice were intraperitoneally administrated every day with Lycorine at the dose of 20 mg/kg/day, or with DMSO as solvent control group. The beginning day of Lycorine administration was defined as day 0. Mice were continually observed and their tumor weight and volume were calculated until they were sacrificed at day 35, which meant the day of experiment ending was 47 days later after the beginning of cell inoculation.

### Patient-derived xenograft model

This assay was performed as described previously with few modifications [40]. Briefly, the patient-derived cells were injected subcutaneously on the right back sides of the mice (5 × 10^6^ cells per mouse). After the tumors reached about 100 mm^3^, we removed them from the mice and dissected them into 30 little pieces, equally. Then these 30 little tumor pieces were subcutaneously transplanted into the right back sides of nude mice after anaesthetized by Afferden, randomly. After the tumors reached about 100 mm^3^, mice were divided into 3 groups and received intraperitoneal injection either with DMSO or Lycorine (10 mg/kg/day per mouse and 20 mg/kg/day per mouse) every day for 14 days. During the administration of Lycorine, the body weight and the tumor size of the mice were monitored every 2 days. Mice were continually observed and their tumor weight and volume were calculated until they were sacrificed.

### Immunohistochemistry staining

Intracranial tumors dissected from the U251-Luciferase cell orthotropic transplantation xenograft model were excised, fixed and embedded in paraffin. To investigate the effect of Lycorine on tumor cells proliferation and apoptosis in vivo, sections (4 μm) were stained with anti-proliferation cell nuclear antigen (Ki-67), GFAP, cleaved caspase-3, p-EGFR and MMP9. Images were obtained with Leica microscope (Leica, DM4000b). The results were analyzed using Image-Pro Plus 6.0 software.

### Statistical analysis

Results were statistically analyzed using the Student’s t test with GraphPad Prism version 4.02 for Windows. All experiments were repeated at least three times. A value of *P* < 0.05 was considered statistically significant.

## Results

### Identifying Lycorine as a novel potential EGFR inhibitor

**Fig. 1:**
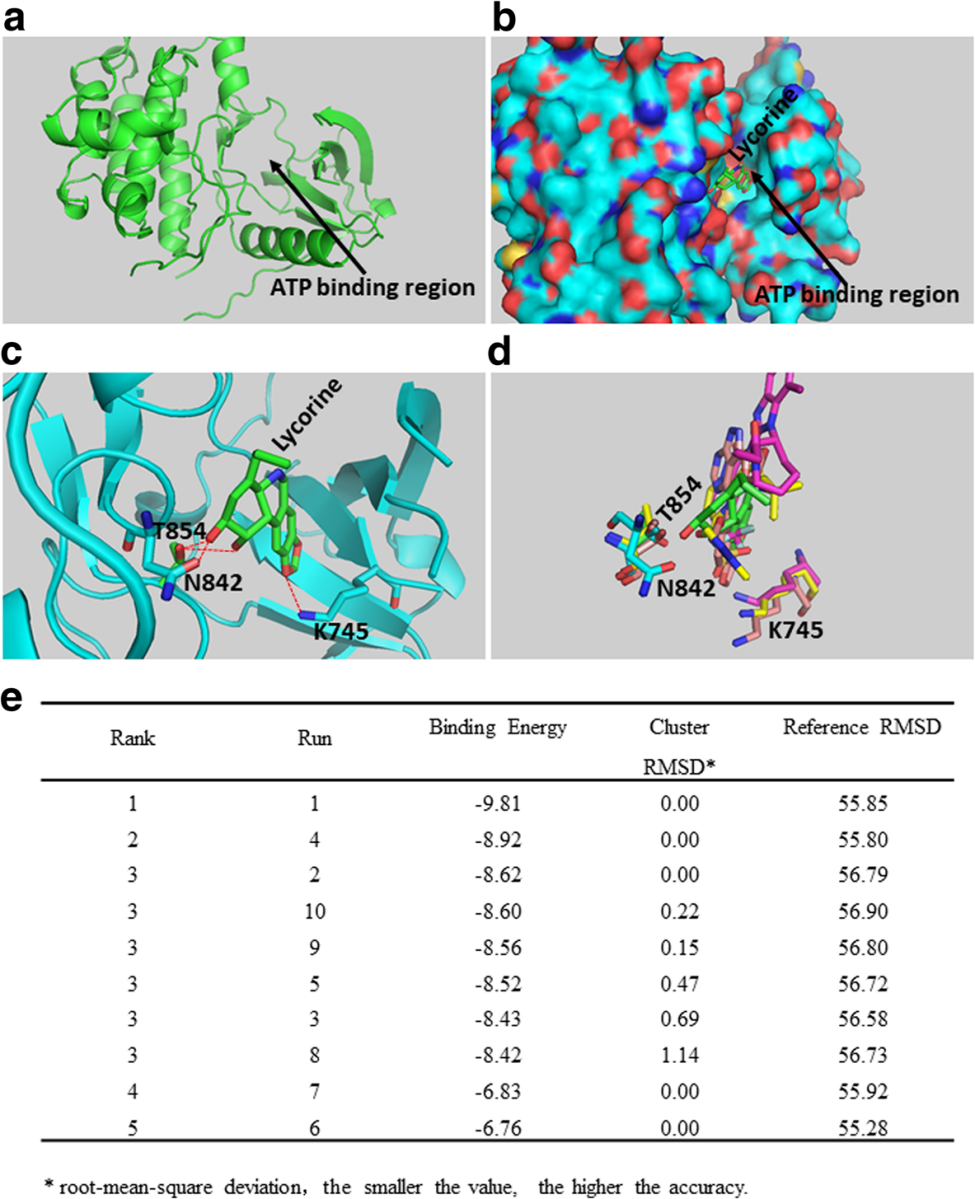
Lycorine interacts with EGFR in molecular docking model. **a** The X-ray crystal structure of EGFR kinase domain, containing an ATP binding region. **b** The binding mode of Lycorine with EGFR kinase domain. Lycorine (green), inserted into EGFR pocket domain (amaranth), which was located at the kinase active site within the ATP binding region. The binding cavity is shown as achromatic surface. **c** Lycorine directly bound with EGFR (696–1022) kinase active sites in the pocket domain via its hydrogen bond interacting to Asn842 (N842), Lys 745 (K745) and Thr854 (T854) in the docking structure. **d** 10 combining conformations (the green one and other amaranth 9) acquired from the flexible docking model between Lycorine and EGFR. **e** The binding free energy with root-mean-square deviation (RMSD) of 10 combining conformations. The smaller the RMSD value, the higher the accuracy

For identifying whether Lycorine is a novel potential EGFR inhibitor for cancer therapy, we downloaded the X-ray crystal structure of EGFR kinase domain from the Protein Data Bank (PDB ID: 5FED, EGFR kinase domain in complex with a covalent aminobenzimidazole inhibitor), and AutoDock Toolkit (ADT) software package was employed to perform the molecular docking assay. Fig. 1a showed the X-ray crystal structure of EGFR kinase domain, which contained an ATP binding region. From Fig. 1b it could be concluded that Lycorine (green), inserted into EGFR pocket domain (amaranth), which was located at the kinase active site within the ATP binding region, thus may destroy the kinase activity of EGFR (Fig. 1b). Fig. 1d showed the flexible docking model between Lycorine and EGFR acquired 10 combining conformations (the green one and other amaranth 9), and Fig. 1e showed each binding free energy with their root-mean-square deviation (RMSD). The first combining conformation (Run 1) ranked the most accurate and reasonable binding model because Run 1 had the lowest binding energy and the minimal cluster RMSD value (Fig. 1e). Lycorine was found to be directly bound with EGFR (696–1022) kinase active sites in the pocket domain, via its hydrogen bond interacting to Asn842 (N842), Lys 745 (K745) and Thr854 (T854) in the docking structure (Fig. 1c). Besides, EGFR (696–1022) domain retained Lycorine in its ATP binding pocket through 3 different interactions: the hydroxide radical of the T854 lateral chain connected to the two hydroxyl hydrogen bonds of Lycorine’s C-ring; the carbonyl of the N842 lateral chain connected to the hydroxide radical of Lycorine’s C-ring; and the _3_HN^+^ of the K745 lateral chain connected to the oxygen atom of Lycorine’s dioxolane. All the above results strongly suggest that Lycorine may function as an EGFR inhibitor and competitively inhibit ATP’s binding with EGFR, thus impede EGFR downstream signal kinases’ autophosphorylation.

### Lycorine impairs proliferation, migration and colony formation of GBM cells

**Fig. 2:**
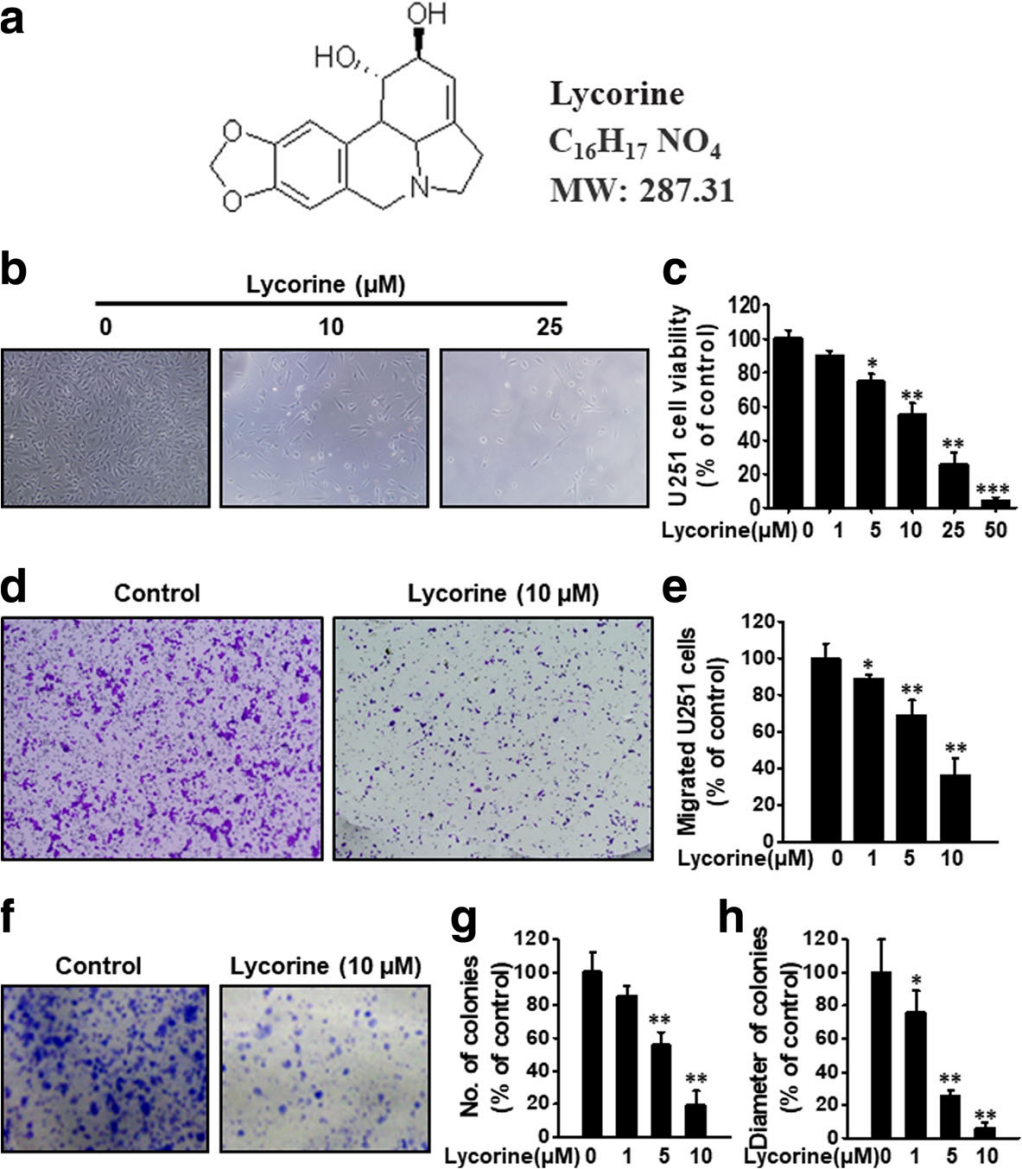
Effects of Lycorine on proliferation, migration and colony formation of GBM cells in vitro. **a** Chemical structure of Lycorine. **b** U251 cells were treated with indicated concentrations of Lycorine (0, 1, 5, 10, 25, 50 μM) for 48 h. Cell viability was assessed by SRB assay (*n* = 3) and photos of the cell morphology were taken by microscope at the light field. **c** Statistical result of Fig. 2b. **d** U251 cells were seeded on the upper chamber of Transwell. After 5 to 7 h incubation with Lycorine (from 0 μM to 10 μM), migrated cells were fixed and stained. The number of migrated cells were calculated. **e** Statistical result of Fig. 2d. **f** Cells were seeded in 6-well plates for 7 days after the treatment of Lycorine in according concentrations and fixed with 4% paraformaldehyde, and stained with 0.2% crystal violet. The statistical results of colony numbers and diameters were presented in **g** and **h**. All data are represented as mean ± S.D. from triplicate wells. *, *p* < 0.05, **, *p* < 0.01, ***, *p* < 0.001, as compared to control

As a small natural product, Lycorine has very simple chemical structure and low molecular weight (Fig. 2a), thus can be easily available to treat cancer cells. To investigate the anti-cancer activity of Lycorine on GBM, a typical malignant GBM cell lines, U251, were subjected to the cell viability assay. Fig. 2b showed Lycorine inhibited cell proliferation in a dose-dependent manner and reduced the number of cultured live cells dramatically, and Fig. 2c statistically demonstrated Lycorine’s inhibition to U251’s cell viability, with an IC_50_ about 10 μM (Fig. 2c). We also performed cell migration assays using U251 cells with highly malignant mobility. Lycorine, in a dose-dependent manner, significantly inhibited U251 cell migration (Fig. 2d), and Fig. 2e showed the statistical results of Fig. 2d. As Fig. 2f showed, Lycorine inhibited colony formation of GBM cells in a concentration dependent manner, and showed a very significant difference compared to the control group when at 10 μM. After being seeded in 6-well plates and colony formatted for 1 week, U251 cells displayed a decreased number of colonies with the increase of Lycorine concentration. Fig. 2g and h show the statistic results of each colony formation assay, according to the diagram of colony numbers (Fig. 2g) and colony diameters (Fig. 2h). Shortly, Lycorine, in a dose-dependent manner, significantly inhibited GBM cell proliferation, migration and colony formation.

### Lycorine exhibits cytotoxicity to GBM cells expressing wild type EGFR and EGFRvIII

**Fig. 3:**
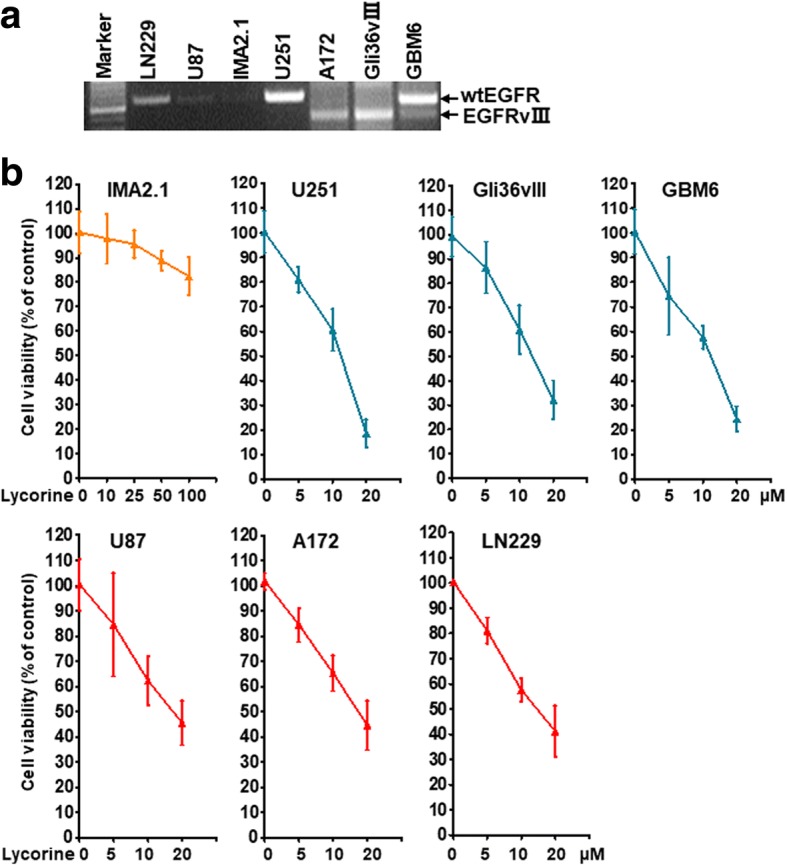
Effects of Lycorine on cell viability of GBM cells holding different EGFR status. **a** Expression of EGFR mRNA on different GBM cell lines. Surface EGFR expression on glioma cell lines (U87, wild type EGFR; LN229, wild type EGFR amplification, U251, wild type EGFR amplification, A172, EGFRvIII mutant, Gli36vIII, EGFRvIII mutant, GBM6, wild type EGFR and EGFRvIII co-existing) and a healthy normal human IMA2.1 astrocytes was monitored by RT-PCR. **b** Lycorine suppresses GBM cell’s proliferation independent of EGFR mutation status but dependent of EGFR expression level. The cell viability assay stained by SRB was performed as described in Methods. All data are represented as mean ± S.D. from triplicate wells

The aforementioned results suggest that Lycorine inhibits the proliferation of U251 cells. Furthermore, we questioned if Lycorine had ideal selective effects between different GBM cells holding different EGFR mutations as well as healthy normal human IMA2.1 astrocytes. 6 kinds of cell death that induced by Lycorine to GBM cells was examined. These 6 cell lines, including U87 (wild type EGFR), LN229 (wild type EGFR amplification), U251 (wild type EGFR amplification), A172 (EGFRvIII mutant), Gli36vIII (EGFRvIII mutant), and GBM6 (wild type EGFR and EGFRvIII co-existing), were all utilized to conduct the cell viability assay. The expression level of EGFR mRNA was confirmed by RT-PCR (Fig. 3a). SRB assay results clearly showed that the half maximal inhibitory concentration for Lycorine inhibition of GBM cellular proliferation was approximately 10–20 μM, while that of normal human IMA2.1 astrocytes were much more than 100 μM (Fig. 3b). In other words, Lycorine was more toxic to GBM cells than to normal brain tissue cells thus can be considered possessing unique selectivity to treat GBM. Moreover, although Lycorine could inhibit all the 6 cell lines of GBM proliferation, its inhibition mode on the 6 GBM cells was different obviously. No matter wild type EGFR or EGFRvIII, the higher expression level those cells harbored, the greater inhibition efficiency presented. For example, for U251, Gli36vIII and GBM6 cells, they all had a higher EGFR or EGFRvIII expression, thus they were seemly more sensitive to Lycorine. At the dose of 20 μM their cell viability reduced to 20% compared with control (Fig. 3b, the upper panel). Situation was different for the other 3 cell lines, U87, A172 and LN229 possibly because they had lower expression level of EGFR or EGFRvIII, thus were not so susceptive to Lycorine, compared with the former 3 GBM cell lines. At the same dose of 20 μM their cell viability reduced to about 40%, relatively much higher than 20% (Fig. 3b, the below panel). In conclusion, results of Fig. 3b suggested that the inhibition effect of Lycorine to GBM cells were correlated with the expression amount of EGFR, no matter wild type EGFR, or EGFRvIII, or other EGFR mutants, thus Lycorine could be considered a candidate to overcome different EGFR mutation status in treating GBM.

### Lycorine suppresses EGF-induced EGFR signaling pathway

**Fig. 4:**
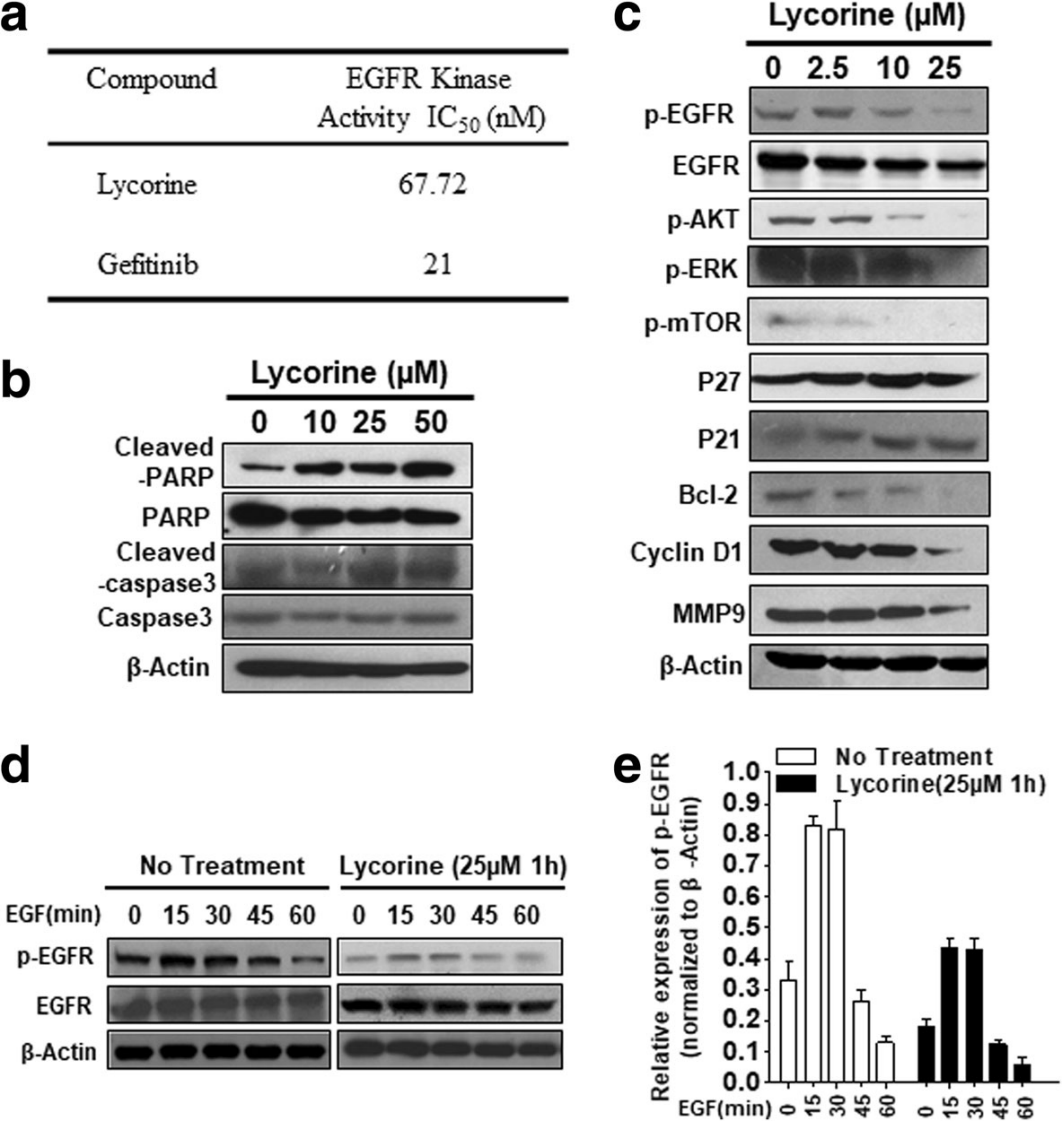
Lycorine’s suppression effects on EGF-induced EGFR signaling pathway. **a** In vitro EGFR kinase inhibition by Lycorine. Half maximal inhibitory concentration (IC_50_) values of Lycorine and positive control Gefitinib. **b** U251 cells were incubated with various concentrations of Lycorine (from 0 μM to 50 μM) for 48 h. Effects on the expression of CL–caspase 3 and PARP were determined by Western blotting. Human β-actin served as a loading control. **c** Suppression by Lycorine of EGFR phosphorylation and its downstream AKT, ERK, mTOR, p27, p21, Bcl-2, Cyclin D1, MMP9 in U251 cell lines. For detecting the phosphorylation of EGFR and its downstream signaling pathways, U251 cells were pretreated with 100 ng/mL human recombinant protein EGF for 6 h, then treated with Lycorine for indicated concentration for another 24 h; and cell lysates were subjected to Western blotting analysis with indicated antibodies. **d** U251 cells exposed to 100 ng/mL human recombinant protein EGF for indicated time points (0, 15, 30, 45 and 60 min) then followed with 25 μM Lycorine for 1 h or with no treatment. The expression of EGFR and p-EGFR were detected by western blotting. **e** Statistical result of Fig. 4d

According to the aforementioned molecular docking suggestions (Fig. 1), the EGFR kinase assay was carried out in the presence of Lycorine or the well-known EGFR protein kinase inhibitor Gefitinib. As shown in Fig. 4a, the kinase activity inhibition IC_50_ for Gefitinib was nearly 21 nM, which is consistent with previous report [41]. The inhibition IC_50_ for Lycorine was about 68 nM (Fig. 4a), suggesting Lycorine directly inhibited the kinase activity of EGFR at a concentration comparable to classical EGFR kinase inhibitor. Then we treated U251 cells with Lycorine to induce apoptosis and western blot analysis was conducted. Clear cleavages of PARP and caspase-3 occurred and suggested that Lycorine suppressed GBM cell growth through its pro-apoptotic effects (Fig. 4b). Next, we checked the effect of Lycorine on EGF-induced EGFR phosphorylation and EGFR protein level. After EGF’s induction for 6 h and then followed Lycorine’s treatment for another 24 h, Lycorine reduced EGF-induced EGFR phosphorylation in a dose-dependent manner. Lycorine at 25 μM fully blocked EGFR phosphorylation. Meanwhile, the expression levels of p-AKT and p-ERK decreased accordingly in the same manner as p-EGFR, while the total amount of EGFR proteins were also declined. Accordingly, some other oncogenic proteins such as p-mTOR, Bcl-2, Cyclin D1 and MMP9 were all down-regulated by Lycorine and some tumor suppressors including p21 and p27 were up-regulated (Fig. 4c). Generally EGF induces EGFR phosphorylation with a fast kinetic so that EGFR phosphorylation peaks within about 1 h then decreases because the activity of tyrosine phosphatases and because down-regulation of EGFR [42]. To more accurately distinguish the inhibition model of Lycorine on EGFR and EGFR’s phosphorylation, we exposed cells to Lycorine only for short period (25 μM, 1 h) and then made a kinetic of EGFR phosphorylation in the presence or absence of EGF for indicated time points such as 0, 15, 30, 45 and 60 min. Results were shown in Fig. 4d and e. After EGF induction, p-EGFR level was significantly up-regulated within 30 min then reduced within 60 min (Fig. 4d, No Treatment panel). Accordingly, 25 μM Lyrorine’s treatment for a short period further aggregated p-EGFR reducing process while the total expression level of EGFR had no obvious change (Fig. 4d, Lycorine panel). The statistic results in Fig. 4e illustrated the apparent difference between EGFR and p-EGFR under the treatment of Lycorine. Briefly, Lycorine decreases EGFR phosphorylation for short treating time whereas decreases both EGFR and p-EGFR for long treating time (Fig. 4c, d and e). All these results confirm the fact that Lycorine inhibits EGFR and its downstream signaling pathways.

### Lycorine binds to EGFR, inhibits EGF-activated EGFR phosphorylation and exhibits an EGFR-dependent manner to suppress GBM cells proliferation

**Fig. 5:**
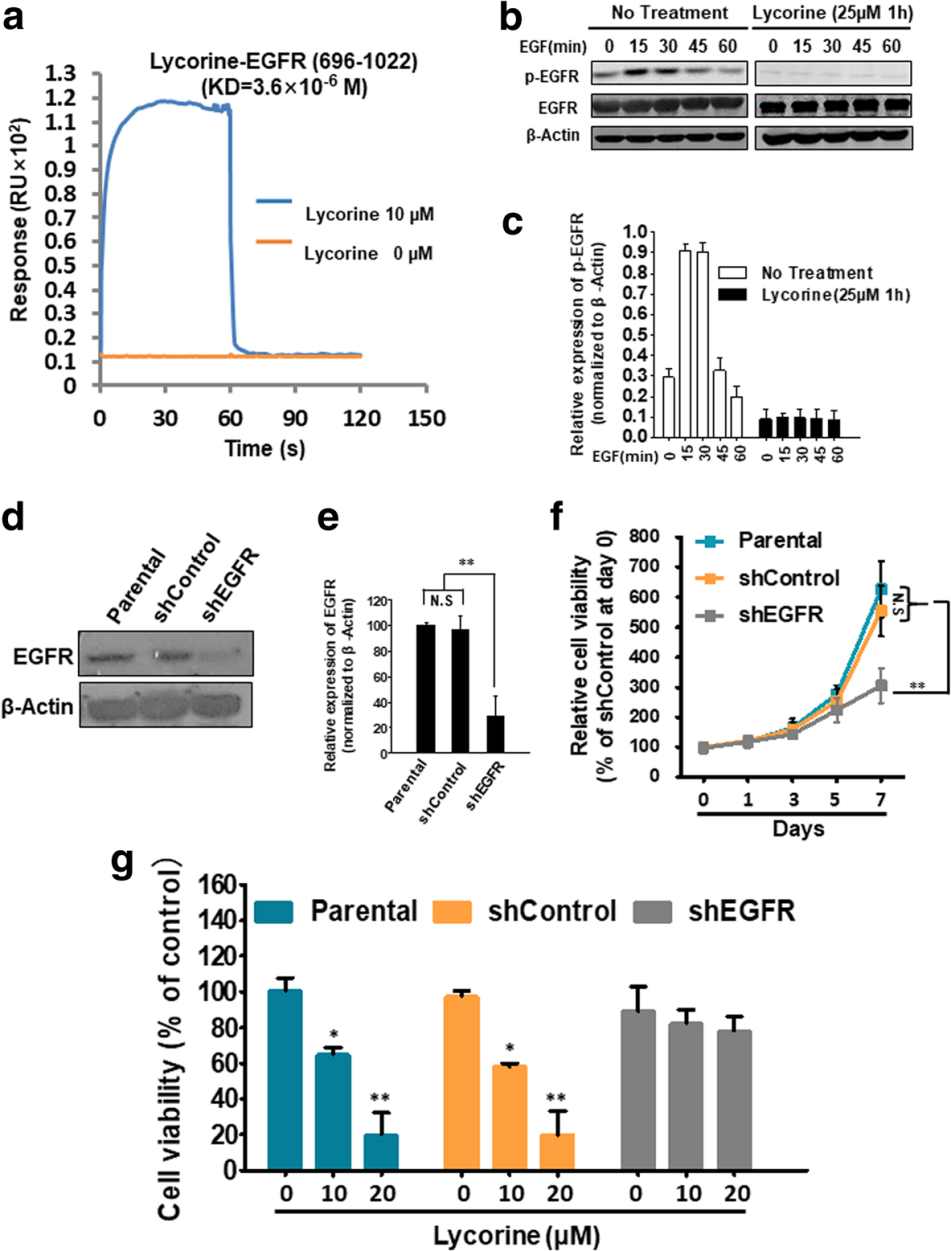
Lycorine binds to EGFR, inhibits EGF-activated EGFR phosphorylation and exhibits an EGFR-dependent manner to suppress GBM cells proliferation. **a** Biacore assay to reveal the SPR analysis of the binding between Lycorine and EGFR (696–1022) domain. The purified EGFR (696–1022) protein was immobilized on an activated CM5 sensor chip. Lycorine was then flowed across the chip. **b** U251 cells were pretreated with or without 25 μM Lycorine for 1 h then followed by 100 ng/mL EGF treatment for 0, 15, 30, 45 and 60 min (Lycorine was maintained during the EGF-treated time course), and EGF-dependent EGFR phosphorylation was measured by western blotting. **c** Statistical result of Fig. 5b. **d** After successful construction of stable U251 shEGFR cells, the knockdown efficiency of EGFR protein was detected by Western blotting in Parental (normal U251 cells), shControl and shEGFR stably constructed U251 cells, respectively. **e** Statistical result of Fig. 5d. **f** Parental (normal U251 cells), shControl and shEGFR stably constructed U251 cells were seeded in 96 well plates for indicated days and cell viability was assessed by SRB assay.**g** Statistic result of cell proliferation when EGFR was interfered by shRNA. Parental (normal U251 cells), shControl and shEGFR stably constructed U251 cells with shRNA were treated with indicated concentrations of Lycorine (0 μM, 10 μM and 20 μM) for 48 h and cell viability was detected by SRB assay. All data are represented as mean ± S.D. from triplicate wells. *, p < 0.05, **, p < 0.01, as compared to control

To further dig out the mechanistic understanding of Lycorine’s inhibition on EGFR, we purified GST-tagged EGFR (696–1022) region in line with our molecular docking result and then subjected it to the Biacore platform. The Biacore assay was utilized to evaluate the binding between Lycorine and EGFR under the principle of surface plasmon resonance (SPR), and the result verified that there was indeed a complex between Lycorine and EGFR. Lycorine interacted directly with EGFR (696–1022). The RU values evaluating Lycorine’s binding to immobilized EGFR demonstrated a dose-dependent manner. Lycorine at 10 μM exhibited significant positive signals while Lycorine at 0 μM almost with no reaction. And the determined equilibrium dissociation constant (KD) between Lycorine and EGFR (696–1022) was about 3.6 μM (KD = 3.6 × 10^− 6^ M) (Fig. 5a). Considering Lycorine direct binds to EGFR (696–1022) and competitively occupies ATP binding pocket of intracellular EGFR region, we speculate that Lycorine may throughout block EGFR autophosphorylation within this tyrosine kinase domain. Hence we treated cells with Lycorine initially and then stimulated cells with EGF. Results showed that even if cells were stimulated by EGF, the amount of p-EGFR was still very faint under Lycorine pretreated groups (Fig. 5b and c, Lycorine panel) while in the No Treatment group, p-EGFR could be significantly induced to a high expression level with a normal kinetic time course (0, 15, 30, 45 and 60 min) (Fig. 5b and c, No Treatment). This results can be explained that when Lycorine enters the cytoplasm, binds with intracellular EGFR (696–1022) domain and occupies the ATP binding pocket of intracellular EGFR and blocks the essential binding process of ATP and EGFR for EGFR’s auto-activated phosphorylation. Thus the amount of p-EGFR in the No Treatment group are much higher than that in the Lycorine pretreated group. In conclusion, our findings prove that Lycorine inhibits EGF-activated EGFR kinase activity.

Lycorine’s interaction and inhibition on EGFR casts our hypothesis whether Lycorine’s antitumor activity depends on EGFR expression. Herein we measured if abolishing EGFR expression by shRNA altered Lycorine toxicity on GBM U251 cells. The knockdown extent of EGFR expression was illustrated in Fig. 5d. Compared with the parental and shControl group, shEGFR group reduced its EGFR expression by nearly 70% (Fig. 5e). The in vitro growth rate of U251 shControl and shEGFR was also measured (Fig. 5f). Long-term knockdown of EGFR indeed decreased GBM cell viability at day 7 because EGFR was an oncoprotein for cancer cell proliferation. However, from day 0 to day 5, no obvious inhibition effects were observed between the shControl and shEGFR, which meant RNA-interfered EGFR might need a long time to exhibit its inhibition on cell proliferation. To avoid RNA-interfered EGFR’s influence on Lycorine, the subsequent cell viability assay was conducted by treating GBM cells with Lycorine for 48 h of a comparably short time, before the time point of day 5 to refrain from EGFR’s long-term knockdown in decreasing cell growth, and the results were shown in Fig. 5g. Short-term EGFR knockdown ablated the ability of Lycorine’s treatment to hinder cell proliferation (Fig. 5g). In parental and shControl groups, EGFR expression was normal so Lycorine showed significant inhibition effects on cell proliferation (Fig. 5g, blue and orange columns). However, Lycorine could not show its obvious inhibition even at the high dose of 20 μM when EGFR was knocked-down, which suggested that Lycorine’s inhibition on cell proliferation was dependent on EGFR expression in vitro (Fig. 5g, gray columns). All these findings suggest that EGFR might be a critical and direct target of Lycorine in GBM cells.

### Lycorine inhibits U251-luc intracranially orthotopic tumor growth in vivo

**Fig. 6:**
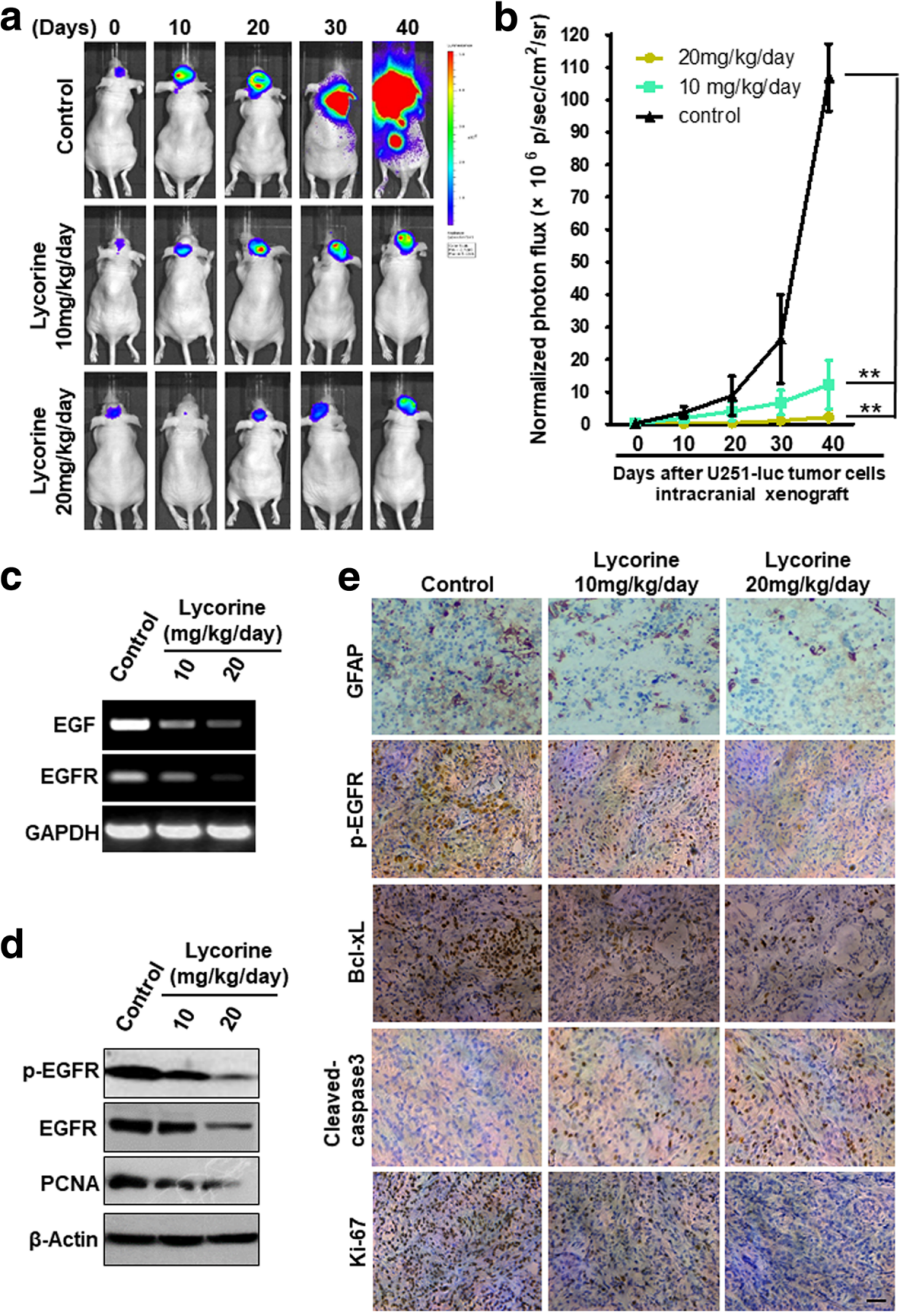
Lycorine inhibits U251-luc orthotopic tumor growth in vivo. **a** Tumor growth in the orthotopic intracranial cavity over a 40-day period was detected by bioluminescence analysis every 10 days. **b** Quantitative analysis of growing cells in brain bioluminescence analysis every 10 days. The means and 95% confidence intervals (error bars) are presented;***, *P* < 0.001, **, *P* < 0.01. *P* values were calculated using a two sided Student’s t test. p/sec/cm^2^/sr = photons/ second/cm^2^/steradian. The inhibitory effect of Lycorine on EGFR signaling pathway in U251-luc orthoropic tumor growth model were sectioned and probed with human EGF, EGFR primers (**c)** and anti-human p-EGFR, EGFR, PCNA antibodies (**d)**. Human GAPDH was served as a mRNA loading control for Fig. 6c. Human β-actin was served as a protein loading control for Fig. 6d. **e** U251-luc orthoropic tumor sections were processed for immunohistochemical analysis to detect human GFAP, p-EGFR,Bcl-xL, cleaved-caspase 3, and Ki-67. Representative images are shown. Brown color indicates positive cells. Scale bar = 30 μm

The orthotopic transplantation tumor model is widely used to imitate the real clinical situation of cancer progression in drug research. In an attempt to mimic human disease to the maximum extent, we evaluated Lycorine’s chemotherapeutic potential on U251 orthotopic tumor growth model in vivo*.* Briefly, a luciferase-expressing U251 cell line (U251-luc) was established by stably transfected with luciferase-expressing plasmids. After injected stereotactically into the mice intracranial right frontal lobe of adult nude mice brains from day 0 to day 40, U251-luc cells exhibited bioluminescence which could be traced by photon flux indexes to represent the tumor sizes using the IVIS 2000 Luminal Imager system. Mice were divided into 3 groups (*n* = 10 per group) and treated with Lycorine at 10 mg/kg/day or 20 mg/kg/day or vehicle control. Tumors in the whole body of each mouse were imaged by IVIS every 10 days to determine local tumor growth and tumor cells dissemination. As shown in Fig. 6, Lycorine evidently impaired the U251-luc orthotopic xenografts in tumor-bearing mice. In the control group, bioluminescence was detected in the whole parts of mice cranial cavity (Fig. 6a) and increased remarkably with day number increase (Fig. 6b). Treatment with Lycorine statistically reduced the photo flux indexes (Fig. 6b). Administration of 20 mg/kg/day of Lycorine almost completely blocked tumor growth. The average normalized photon flux of the 10 mg/kg/day Lycorine treated group and 20 mg/kg/day Lycorine treated group was (2.14 ± 0.51) × 10^6^p/sec/cm^2^/sr and (13.57 ± 1.28) × 10^6^p/sec/cm^2^/sr, respectively, while that of control group was (106.03 ± 3.43) × 10^6^p/sec/cm^2^/sr (Fig. 6). At day 40 the nude mice were sacrificed and the orthotopic xenografts were stripped for molecular biological detection. When exploring the signal pathways after Lycorine administration in vivo by RT-PCR, western blotting and immunohistochemistry analysis, we found the expression of EGF and EGFR decreased in both mRNA (Fig. 6c) and protein level (Fig. 6d), while the expression of p-EGFR, Bcl-xL and Ki-67 decreased, compared to the control group. As an intermediate filament protein considered to be the best astroglial marker, GFAP also reduced after Lycorine treatment. Conversely, the apoptotic marker Cleaved caspase 3 was up-regulated (Fig. 6e). Together, these in vivo findings were in agreement with our in vitro results and indicated that Lycorine therapeutically suppressed GBM tumor growth in intracranially orthotopic xenograft model through suppressing the EGFR signaling pathway.

### Lycorine’s inhibition on GBM growth is dependent on EGFR in vivo

**Fig. 7:**
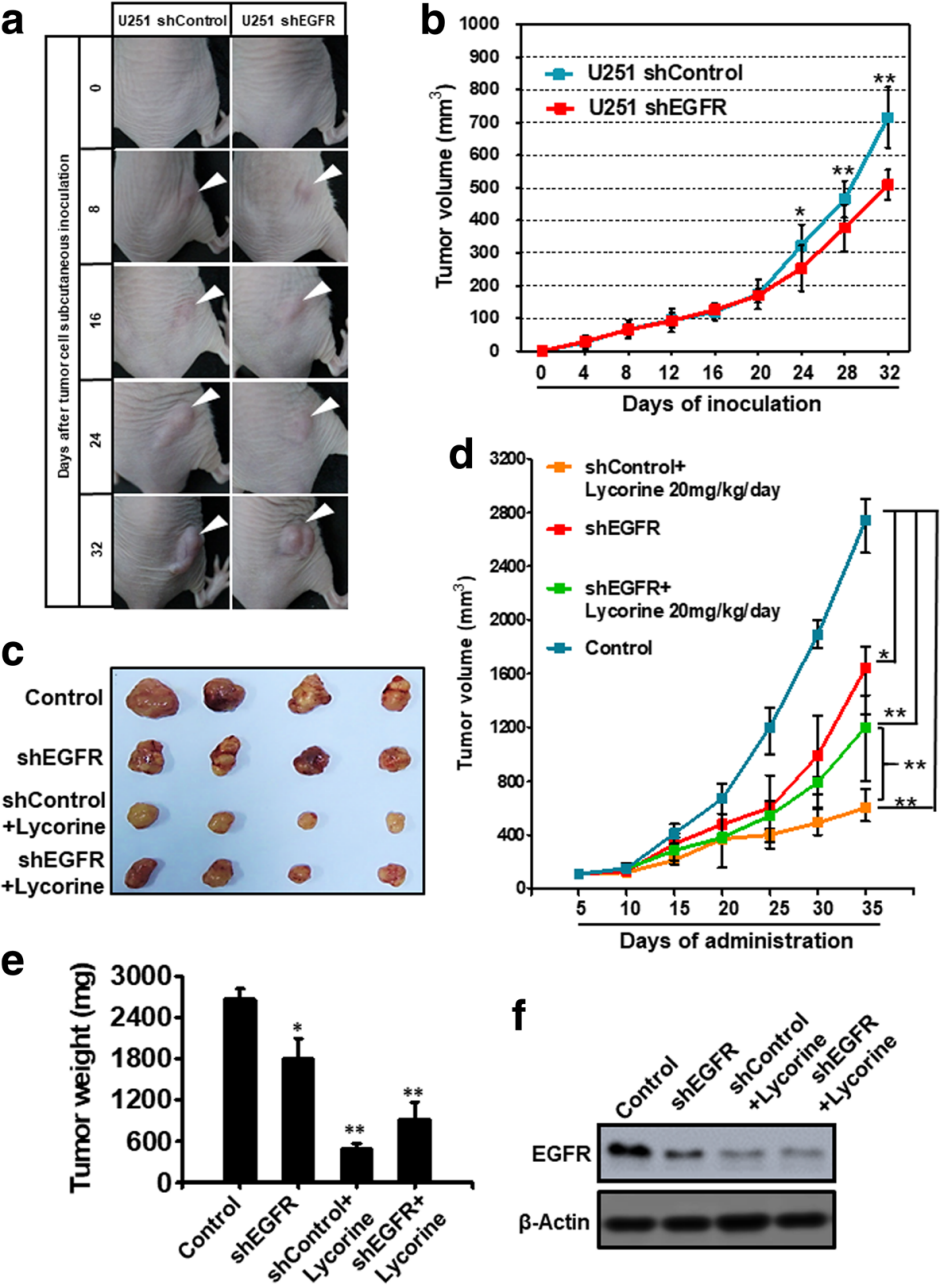
Lycorine’s inhibition on GBM growth is dependent on EGFR in vivo*.* **a** Photos of tumor-bearing nude mice and their xenografts at indicated days of U251 shControl and shEGFR after inoculation. White triangle indicates the tumors that grow subcutaneously in mice right backs. **b** The growth curve of U251 shControl and shEGFR tumor volume after their inoculation from day 0 to day 32. **c** Representative images of tumor tissue in control, shEGFR, shControl+Lycorine 20 mg/kg/day and shEGFR+Lycorine 20 mg/kg/day groups. **d** The tumor volume of 4 groups was shown through growth curve (*n* = 4, **, P < 0.01). **e** The statistic result of tumor weight in control, shEGFR, shControl+Lycorine 20 mg/kg/day and shEGFR+Lycorine 20 mg/kg/day groups. The means and 95% confidence intervals (error bars) were presented (n = 4, **, P < 0.01). **f** Dissected tumor tissues were extracted protein and subjected to Western blotting analysis and the expression of EGFR was detected in 4 groups

Like Lycorine’s in vitro effects on shEGFR cells that have been revealed in Fig. 5, the in vivo subcutaneous xenograft assay was also performed to assess EGFR disturbance on Lycorine’s inhibition on GBM growth. Firstly we tested the growth rate of U251 shControl and shEGFR in vivo without Lycorine treatment. Photos in Fig. 7a showed the phenotype of tumor-bearing nude mice and their xenografts at indicated days, and the growth curve in Fig. 7b elucidated the detailed data of U251 shControl and shEGFR after their inoculation from day 0 to day 32 with a final tumor volume of 703 ± 2.19 mm^3^ and 512 ± 11.04 mm^3^, respectively. There was no significant difference between shControl and shEGFR until their inoculation after day 24. But from day 24 to day 32, knockdown of EGFR indeed reduced GBM tumor growth in vivo. All tumors reached to a volume of nearly 100 mm^3^ at day 12, which meant at an early stage of in vivo experiment, the growth rate of shControl and shEGFR were the same. This in vivo result was consistent with in vitro results of Fig. 5f. Likewise, we determined the time point of day 12 applicable to initiate Lycorine administration to measure whether abolishing EGFR expression by RNA interference could alter Lycorine’s toxicity on GBM xenografts in vivo or not. Then we conducted the in vivo subcutaneous xenograft assay again under the treatment of Lycorine. The tumor size, volume and weight of subcutaneous xenografts were demonstrated in Fig. 7c, d and7e. When EGFR was knocked-down by stable shRNA, even at the largest dose of 20 mg/kg/day, Lycorine still faded its severe inhibition on GBM growth when compared with the control group (Fig. 7c, d and e). It was safe to infer that EGFR’s deprivation (shEGFR group) reduced GBM growth compared to the control group because EGFR exerted as a promoting factor for many caner types, especially for GBM. However, as EGFR expression was reduced by EGFR shRNA, Lycorine’s inhibition on GBM growth also declined dramatically (Fig. 7d, green curve, compared with the orange curve of shControl group treated with 20 mg/kg/day of Lycorine, ** *P* < 0.01). Fig. 7f verified EGFR expression level after Lycorine administration and shRNA interfering in vivo. Lycorine down-regulated the expression level of EGFR in vivo. Interestingly, in our experiment result of Fig. 7f, Lycorine could reduce EGFR expression to a more severe extent than EGFR shRNA do. This phenomenon might partially explain why the tumor volume in the shControl group treated with 20 mg/kg/day was much smaller than that in the shEGFR group (Fig. 7c, d and 7e). And Lycorine at the dose of 20 mg/kg/day still exhibited some inhibition effect on tumor growth even in the shEGFR group. This might be due to the possible reason that Lycorine may have some other pharmacological targets besides EGFR, which meant EGFR was not the only one target of Lycorine in vivo. Another explanation might be that the EGFR shRNA used in our experiment was not efficient enough to completely disappear EGFR expression, thus even just a litter remaining EGFR might contribute to Lycorine’s inhibition on tumor growth in the shEGFR xenografts. Anyhow, these results provide rationale evidence that Lycorine’s inhibition on EGFR also occurred in vivo and this inhibition was dependent on EGFR.

### Lycorine retards the growth of patient-derived GBM tumor xenografts

**Fig. 8:**
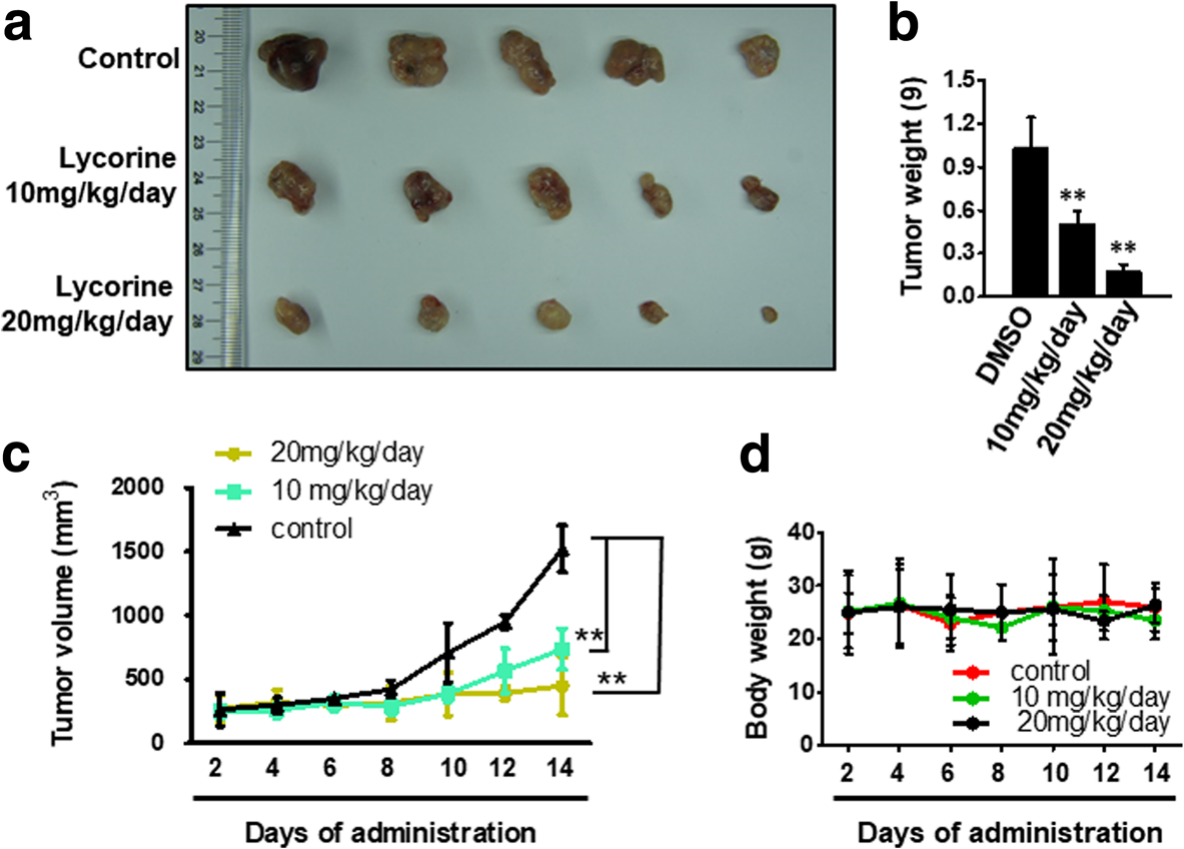
Lycorine hinders the growth of patient-derived GBM tumor xenografts. **a** The patient-derived *in sute* tumor cells were injected subcutaneously into the nude mice, which was operated according to the ways described in Methods. After mice sacrificed, tumors were removed and images taken with a Nikon camera. **b** Statistic results of quantitative tumor weight analysis of subcutaneous lesions after mice sacrificed. **c** Statistic results of quantitative analysis of tumor volume with the day’s growing in mice every 2 days. **d** Effect of Lycorine on mouse body weight. Lycorine did not affect the body weight of mice when recorded every2 days. The means and 95% confidence intervals (error bars) were presented (** P < 0.01)

Finally, we clinically examined the effect of Lycorine on GBM tumors. It has been widely accepted that patient-derived tumor xenograft models can be utilized as an ideal drug-screening tool for many kinds of cancer therapy including therapy of GBM [40, 43]. We employed a patient-derived GBM cell line, which was primarily separated from a high-grade patient’s in situ tumor of glioma in Xianning central hospital, the first affiliated hospital of Hubei University of Science and Technology (Xianning China), to test if Lycorine could be clinically beneficial. First, the SRB assay was performed to identify the effects of Lycorine on this patient-derived GBM cell line. Within our expectation, Lycorine inhibited cell proliferation of this cancer cell line in a dose-dependent manner (Data not shown). Then, we injected this cancer cell line into nude mice to establish the patient-derived GBM subcutaneous tumor xenograft model. Mice were divided into 3 groups (*n* = 10 per group) and treated with Lycorine at 10 mg/kg/day or 20 mg/kg/day or vehicle control. At the day 14, mice were sacrificed and the tumor xenograft of each mouse was dissected (Fig. 8a). And the tumor weight of each lesion was calculated. Lycorine significantly retarded the growth of tumor volume (Fig. 8b). The average tumor volume of control group was 1621 ± 28 mm^3^, whereas tumor size in Lycorine -treated group was 734 ± 56 mm^3^ for 10 mg/kg/day group and 403 ± 64 for 20 mg/kg/day group, respectively. And statistical results showed a significant difference between the drug-treated groups and the control group (Fig. 8c), especially for the 20 mg/kg/day group, the tumor burden of each mouse almost ceased to grow following the administration of Lycorine (Fig. 8c). At the same time, treatment of Lycorine at the given concentration had little toxic effect on the body weights of the Lycorine -treated mice at the curative dose (Fig. 8d), which was consistent with the results of our previous report [23].

## Discussion

Despite advances in multimodality therapies such as surgery, radiotherapy, and chemotherapy, glioblastoma remains the most aggressive primary brain malignancy with an average post-diagnostic survival of just over 14 months [44]. Considering the extremely poor outcome for patients, increasing the magnitude of chemotherapy standard procedure can improve overall survival in GBM. EGFR contributes to the differentiation, proliferation, survival, migration and invasiveness of cancer cells and increases tumor angiogenesis [2]. Aberrantly activated EGFR affects a wide range of human cancers, particularly lung cancer, colorectal cancer, pancreatic cancer and glioblastoma. Among all these cancer types, glioblastoma has the highest rate of EGFR gene alteration. EGFR and the mutant EGFRvIII are major focal points in current concepts of targeted cancer therapy for GBM, so they are considered to be responsible for tumor initiation, propagation, recurrence, and chemo- and radio-resistance [45, 46]. There are several treatments available [47], including monoclonal antibodies like cetuximab or small molecule inhibitors like Gefitinib, and a vaccination called rindopepimut was even developed to be administered to EGFRvIII-positive tumors [48–50]. However, glioma cells treated with those agents often show resistance mechanisms [51]. Diverse combination drug strategy are already in clinical trials but there is still urgent need to develop novel effective therapeutics.

Many challenges remain to be addressed to effectively target EGFR-dependent GBM. Currently available EGFR inhibitors often fail to achieve adequate inhibition of EGFR in tumors due to sub-optimal brain distribution. GBM are enriched for EGFRvIII mutations in the extracellular domain (ECD) of EGFR, which are refractory to the first-generation EGFR kinase inhibitors, such as Gefitinib and Erlotinib [51]. So far, the vast majority of combination studies included EGFR- or EGFRvIII-specific agents together with radiation or broad alkylating reagents like temozolomide. Although a few of these agents are already approved by the Food and Drug Administration for different cancer types, e.g., cetuximab for colorectal cancer, gefitinib and erlotinib for non-small cell lung cancer, unfortunately, none are approved for glioma treatment yet [52].

In the present study, we investigated the role of Lycorine in the tumor growth as a possible drug candidate for GBM. Lycorine effectively down-regulated EGFR signaling pathway, both at mRNA and protein expression levels. Previously reported studies only focus on targeting EGFRvIII or wild type EGFR. Here we explore whether both wtEGFR and EGFRvIII can be effectively targeted by Lycorine to treat GBM. Lycorine displayed enhanced cytotoxic capability when co-cultured with GBM cells. In detail, cell proliferation, migration, colony formation and cell apoptosis were all responded to Lycorine. Furthermore, Lycorine impaired GBM tumor growth in three different xenograft models (an U251-luc intracranially orthotopic transplantation model, an EGFR stably knockdown U251 subcutaneous xenograft model and a patient-derived xenograft mouse model), dependent on EGFR overall expression. The higher expression level of EGFR that cancer cells harbored, the greater inhibition efficiency that Lycorine displayed. The inhibition effect of Lycorine to GBM cells were solely correlated with the expression amount of EGFR, no matter wild type EGFR or EGFRvIII or other EGFR mutants, suggesting Lycorine can overcome different EGFR mutation status in treating GBM. These findings support drug administration of Lycorine represents a promising clinical strategy to treat GBM.

As most anticancer drugs, Lycorine might probably more efficient when acting on rapidly cycling cells than on slowly dividing ones. And EGFR functions as an important mitogen driving factor in GBM [53, 54]. EGFR downregulation by shRNA indeed reduced GBM cell growth. The possibility that the decreased toxicity of Lycorine on U251 shEGFR may due to slower cycling cells couldn’t be excluded. Thus it was really hard to divide Lycorine’s effects on GBM growth was EGFR-dependent or -independent. However, this would not be a confusion anymore after the revealing of our current research. Lycorine was less toxic on GBM cells in which the expression of EGFR was decreased by stable RNA interference (U251 shEGFR) might be suggestive of a role of EGFR in Lycorine action. However, through in vitro and in vivo EGFR knockdown, we measured the growth rate of U251 shControl and U251 shEGFR to distinguish the growth inhibition was mainly caused by Lycorine treatment or by EGFR downregulation. And both our in vitro and in vivo experiments delicately avoid the complexity because we chose an applicable short time point to conduct Lycorine treatment to exclude EGFR downregulation effects on in vitro GBM cells proliferation and in vivo GBM tumor growth. At least in our current research system, we could confirm that the effects of Lycorine played a leading course when treating GBM cancer, even if EGFR’s knockdown might slow cell cycling. Therefore, it was safe to assert Lycorine acted through an EGFR-dependent pathway in its suppression on GBM.

Lycorine belongs to isoquinoline alkaloids extracted from the perennial medicinal plant Lycoris of Amaryllidaceae genera that widely distributed in China. Many researches have reported Lycorine’s excellent biological activities, including anti-tumor activity. Although Lycorine does not have a defined protein target or action mechanism, it is supposed to be a candidate for clinical application. For instance, a drug containing Lycorine as an effective component has been clinically used in Russian as an expectorant to treat chronic and acute inflammatory processes in lungs and bronchial diseases [55]. Lycorine also promotes hematopoietic stem and progenitor cell niche colonization [56]. As a natural small molecular product, Lycorine holds many advantages such as multi-channel, multi-target and few side effects. Besides, Lycorine exhibits ideal biosafety. Particularly important, Lycorine is an agent that can effectively penetrate the blood-brain barrier (BBB) and doesn’t induce obvious CYP3A4 inhibitory activity [22], which means that GBM primary tumors in the cranial cavity can be easily accessible by Lycorine administration through oral intake or intravenous injection without systemic hepatotoxicity. The reason why GBM still remains difficult to treat despite recent advances in targeted therapy is that the central nervous system is hard for drugs to transport to the cranial cavity because of the BBB. Our findings reveal Lycorine may function as a drug that not only can inhibit EGFR but also can cross the BBB to target intracranial tumors. This drug shows promising effectiveness in GBM orthotopic mouse models as well as in patient-derived xenograft model. Like the latest reported AZD3759, a BBB-penetrating EGFR inhibitor for the treatment of EGFR mutant NSCLC with brain metastases [57], Lycorine may be developed clinically, with the goal of achieving high enough drug concentrations within the CNS. All of these abovementioned properties make Lycorine potential for the pharmacological application for GBM, the most notorious malignancy in human brain. Besides, more detailed factors such as Lycorine’s free concentrations in the blood, cerebrospinal fluid, and brain tissue of Lycorine distribution, need further investigation. Summarily, our data confirms the potential of Lycorine for the treatment of GBM and support its further clinical evaluation in larger trials.

Considering the binding mode of Lycorine with EGFR, our molecular docking results and Biacore analysis elucidated that EGFR (696–1022) domain retained Lycorine in its ATP binding pocket through 3 different interactions and 2 of them were mediated by its C-rings: the first C-ring connected the hydroxide radical of the T854 lateral chain of EGFR (696–1022) domain through two hydroxyl hydrogen bonds of Lycorine; the second C-ring connected the carbonyl of the N842 lateral chain of EGFR (696–1022) domain to the hydroxide radical of Lycorine. Our results are consistent with some previous researches. For example, X-ray structural information of Lycorine in complex with eukaryotic ribosome revealed Lycorine utilized its dioxol-pyrroline group to contact the pocket region in the A-site of the peptidyl transferase center of ribosomes [33]. Another SAR analysis of Lycorine with its intracellular targets elaborated Lycorine’s C1, C2-hydroxyls provided a superior binding pose with the pocket a, the GTP binding site, of its target protein eEF1A [26]. It can be inferred that the C1, C2-hydroxyl rings of Lycorine may be a recognition motif for the binding with its target complex proteins and play critical role for its drug potential. This reminds researches should pay special attention to protect and make full use of this region when developing Lycorine as lead compound drug candidate or when synthesizing Lycorine’s modified derivatives.

Our findings that Lycorine has inhibitory effects on EGFR pathway function exactly an extension mechanism compared with some previous literatures. Lycorine’s mode of action such as its inhibition on protein biosynthesis, its apoptosis-inducing activity, its cell-cycle arresting activity, its anti-proliferative, its anti-invasive properties and its autophagy-promoting activity have already been revealed associated with the JAK, STAT, phospho-Akt, and TCRP1/Akt/mTOR axis. All these molecules are downstream pathway signals of EGFR. That’s why we link the mechanism of Lycorine’s inhibition on GBM to EGFR. On one hand, the existing X-ray structure of Lycorine and EGFR provide virtual structural basis of their interaction. On the other hand, some published literatures which revealed some superficial mechanism of Lycorine’s inhibition on cancer remind us to consider there might be some intrinsic relationship between Lycorine and EGFR, because Lycorine really has influence on EGFR’s downstream signals such as JAK, STAT, AKT and mTOR. Summarily, our current research for the first time provides a direct evidence that Lycorine binds with its intracellular target, EGFR. Therefore, our research contributes great progress in elucidating Lycorine’s pharmacological activity and makes significant sense in understanding Lycorine’s mechanistic drug target.

However, some deficiencies of this present study must be admitted. Firstly, the effective concentration of Lycorine to inhibit EGFR and cure GBM is somewhat high, compared with clinically classical EGFR inhibitors such as Gefinitib, which functions at nanomole level. The fact that Lycorine executives a mocromole concentration to suppress GBM cells may limit its drug clinical applicability. Favorably, Lycorine’s chemical structure is very simple and possesses a typical alkaloids’ tetracyclic skeleton. Therefore, it’s easy to conduct the analysis of structure-function relationship according to its chemical structure. Using Lycorine as a lead compound to synthesis modified derivatives may be a promising direction for novel drug development. And this direction need more extensive research interests and will represent far-reaching value of medical research implications in GBM clinical treatment. Secondly, although our results reveal the direct interaction between Lycorine and EGFR (696–1022) domain and this interaction endows Lycorine’s inhibition on EGF-activated EGFR kinase phosphorylation, further detailed mechanism still wait for future exploration. As typical RTK, EGFR is a membrane-spanning protein with N-terminal extracellular ligand-binding domains to interact with EGF or other ligands, and C-terminal intracellular catalytic domains. After ligands stimuli, EGFR is activated via binding of their extracellular domain elicits RTK oligomerization and activation. Then signals are transduced to the intracellular tyrosine kinase activity domain and EGFR autophosphorylation occurs. Activated auto-phosphorylated EGFR may trigger a number of signaling pathways contributing to tumorigenesis and progression. In our study, we elucidate that Lycorine binds to EGFR and inhibits EGF-activated EGFR phosphorylation through different western blotting results when treating cells using EGF first then followed by Lycorine, or using Lycorine first then followed by EGF. If cells were stimulated by EGF first to induce EGFR kinase activity herein express high level of p-EGFR, then Lycorine could downregulate EGF-induced EGFR phosphorylation and its downstream signals (Fig. 4c and d). And these result also differ two situations when treating cells with long time Lycorine (Fig. 4c) or short time Lycorine (Fig. 4d). If cells were treated with Lycorine initially and then stimulated with EGF, Lycorine could enter the cytoplasm, bind with intracellular EGFR (696–1022) domain and occupy the ATP binding pocket of intracellular EGFR, which might hinder EGFR autophosphorylation, because Lycorine might block the essential binding process of ATP and EGFR for EGFR’s auto-activated phosphorylation. Thus even if cells were stimulated by EGF, the level of p-EGFR was still too low to be detected under Lycorine pretreated groups (Fig. 5b and c). Anyhow, our findings prove that Lycorine inhibits EGF activation of EGFR kinase activity. We may also infer that the extracellular EGF be no inclined to have any relationship with intracellular Lycorine. However, our present study indeed finds Lycorine reduces the mRNA level of EGF and EGFR in vivo (Fig. 6c) and down-regulates both total EGFR and p-EGFR in vitro (Fig. 4c). The intrinsic regulation mechanism between Lycorine and EGF/EGFR is still cryptic. Why Lycorine can affect the transcription of EGF? How Lycorine can reduce the protein expression of EGFR? Whether Lycorine can regulate EGFR’s endocytosis, degradation, cycling, and nuclear translocation and so on? All these detailed mechanisms between Lycorine and EGF/EGFR need to be further explored.

## Conclusions

To sum up, our findings confirm Lycorine inhibits GBM growth through EGFR suppression in terms of the way that Lycorine treatment reduces EGFR expression level and inactive EGFR downstream signaling pathway through direct binding to EGFR. Our research provides a proof-of-principle that targeting the alternatively amplified and mutated EGFR by Lycorine could be used to substitute existing EGFR inhibitors and hinder GBM tumor growth.

## Abbreviations

BBB: blood-brain barrier
CNS: Central nervous system
EGFR: Epidermal growth factor receptor
EGFRvIII: EGFR variant III mutant
GBM: Glioblastoma multiforme
IC_50_: Half maximal inhibitory concentration
MMP9: Matrix metalloproteinase 9
PDB: Protein Data Bank
RTKs: Receptor tyrosine kinases
RT-PCR: reverse transcription-polymerase chain reaction
SPR: surface plasmon resonance
